# ASAP: a machine learning framework for local protein properties

**DOI:** 10.1093/database/baw133

**Published:** 2016-10-01

**Authors:** Nadav Brandes, Dan Ofer, Michal Linial

**Affiliations:** Department of Biological Chemistry, The Alexander Silberman Institute of Life Sciences, The Hebrew University, Jerusalem 91904, Israel

## Abstract

Determining residue-level protein properties, such as sites of post-translational modifications (PTMs), is vital to understanding protein function. Experimental methods are costly and time-consuming, while traditional rule-based computational methods fail to annotate sites lacking substantial similarity. Machine Learning (ML) methods are becoming fundamental in annotating unknown proteins and their heterogeneous properties. We present ASAP (Amino-acid Sequence Annotation Prediction), a universal ML framework for predicting residue-level properties. ASAP extracts numerous features from raw sequences, and supports easy integration of external features such as secondary structure, solvent accessibility, intrinsically disorder or PSSM profiles. Features are then used to train ML classifiers. ASAP can create new classifiers within minutes for a variety of tasks, including PTM prediction (e.g. cleavage sites by convertase, phosphoserine modification). We present a detailed case study for ASAP: CleavePred, an ASAP-based model to predict protein precursor cleavage sites, with state-of-the-art results. Protein cleavage is a PTM shared by a wide variety of proteins sharing minimal sequence similarity. Current rule-based methods suffer from high false positive rates, making them suboptimal. The high performance of CleavePred makes it suitable for analyzing new proteomes at a genomic scale. The tool is attractive to protein design, mass spectrometry search engines and the discovery of new bioactive peptides from precursors. ASAP functions as a baseline approach for residue-level protein sequence prediction. CleavePred is freely accessible as a web-based application. Both ASAP and CleavePred are open-source with a flexible Python API.

**Database URL:** ASAP’s and CleavePred source code, webtool and tutorials are available at: https://github.com/ddofer/asap; http://protonet.cs.huji.ac.il/cleavepred.

## Introduction

The classic approach to annotating residue-level functional properties such as post-translational modification (PTM) sites relies on sequence similarity, augmented by multiple sequence alignments (e.g. HMM profiles in Pfam) (1). Other resources such as PROSITE (2) and ELM (3) provide simple rules for protein ‘signatures’ (4). Rule-based methods suffer from high false positives rates, making them suboptimal for genomic scale retrieval tasks, especially while experimental confirmation remains an expensive bottleneck.

Most properties cannot be reliably represented by simple motifs (e.g. structural disorder). Modern computational methods frequently rely on alternative Machine learning (ML) methods. ML approaches are the state of the art in most non-classic prediction challenges. These methods are applied in community annotation challenges such as Critical Assessment of protein Function Annotation (CAFA) (5,6), and Critical Assessment for Information Extraction in Biology (BioCreAtIvE) (7). ML approaches actually benefit from the growth of available sequences, while ‘brittle’ rule-based methods often fail to cope with the growing variability and quantity of possible annotations and sequences. ML methods have been used for many residue-based predictions such as sorting signals (e.g. SignalP) (8), PTMs (e.g. mammals’ O-glycosylation sites) (9) and immunological features. Importantly, most existing predictors are very specific. For example, phosphorylation sites are predicted separately for eukaryotic, bacterial and yeast proteins, or for specific enzyme families.

Successful applications of residue-level predictions using ML include post translation modification sites (10,11), secondary structure (12,13), disordered regions (14), functional families (15), protein–protein interactions (16) and more. Despite the many ML classifiers used in literature, no generic feature extraction framework or extendable API is available for extracting sequence level properties as learnable features. Most implementations are not designed for general use, but are specialized to each individual framework, preventing their re-use in other applications, even when the derived features are identical. An initial effort in this direction for extracting features from whole proteins is ProFET (17) which showed success in a broad range of classification tasks. ProFET introduced the use of global and local engineered features for classifying neuropeptides (18), thermophile sequences, structural classes and more. However, different types of features and representations are required for residue-level annotation. Thus, in a similar line of thinking, we developed Amino-acid Sequence Annotation Prediction (ASAP), a framework for residue-level ML, including feature extraction, data loading and model training.

We demonstrate ASAP in predicting post-translational proteolytic cleavage sites in precursor proteins by using the framework to train a model called CleavePred. CleavePred predicts cleavage sites for proproteins such as prohormones. The processing proteases of proproteins in Metazoa belong to a diverse family of proteases called Proprotein/Prohormone Convertases (PCs). The unified rule for PCs is the presence of an arginine (R) or a lysine (K) at the first position N-terminally to the proteolytic site (19), though this is by no means sufficient to guarantee cleavage in itself.

The most direct experimental evidence for a cleavage event is by identifying products using tandem mass spectrometry experiments (MS/MS), followed by peptide identification schemes (20), resulting in the many novel peptides identification in recent years (21). For example, NeuroPep database (22) includes over 5000 experimentally identified peptides from ∼500 organisms. Despite this impressive collection, many active peptides remain unidentified due to their small length, altered mass by post-translational modifications (PTM) and poor sequence conservation (18,23,24).

The cleaved products are active peptides that modulate cellular communication in the endocrinal and neuronal systems. Cleavage by PC enzymes usually results in activation of the proprotein, but inactivation of the end product was recorded as well. Major families of peptides produced and activated by PC proteolytic activity include neuropeptides, cytokines, antimicrobial peptides, toxin-like proteins, growth factors and neuroendocrine modulators. However, regulated cleavage by convertases occurs also on GPCR proteins, integrins and membrane receptors. In the human proteome alone over 1000 secretory proteins were proposed as potential substrates for furin, one of the most studied PC enzymes (25). ML methods should narrow the gap between the limited set of validated sites to overlooked substrates with high probability PC regulated cleavage.

In this paper, we focus on using ASAP as a starting point for developing high performance classifiers for any residue-level binary classification task. We illustrate it for the task of identifying proteolytic cleavage sites from other basic residues, and we discuss the results. CleavePred's high precision makes it a promising tool for identifying likely candidates for experimental validation in newly sequenced genomes.

## Methods

### ASAP pipeline

The general problem we are addressing in this research is residue-level prediction (RLP). Namely, predicting functional annotations for individual residues of a sequence. For example, we might want to predict for each residue on a protein whether it is a certain PTM site (e.g. S/T phosphorylation). Predictions can be binary (0 or 1) and probabilistic (e.g. ‘87% probability of being 1’). The framework can easily be adapted to multiclass prediction. To this end, we developed ASAP, a Python framework for feature extraction and ML prediction. ASAP is completely generic, and can be easily applied to any task that involves classifying local sequence properties in proteins.

Applying ASAP to the case study of predicting cleavage sites in protein precursors, we created CleavePred, an ASAP-based model trained to solve the following RLP task: for each candidate residue in the precursor protein, predict whether it is a cleavage site or not. ASAP provides a complete pipeline for data handling, feature extraction, transformation and model fitting. Initial input to ASAP is a dataset of annotated sequences in the ‘lf’ (labeled file) format, with each residue annotated with 0 or 1.

**Figure 1.**
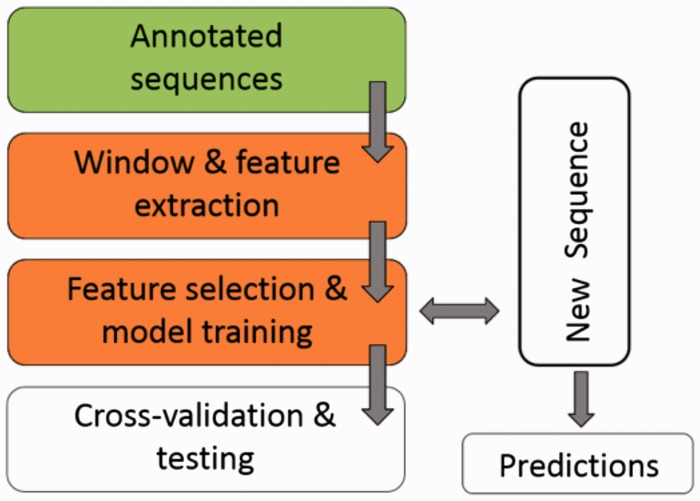
A scheme for the workflow of ASAP. The core of the framework is the ‘Window & feature extraction’ stage. See ‘Methods’ section for details.

**Figure 2.**
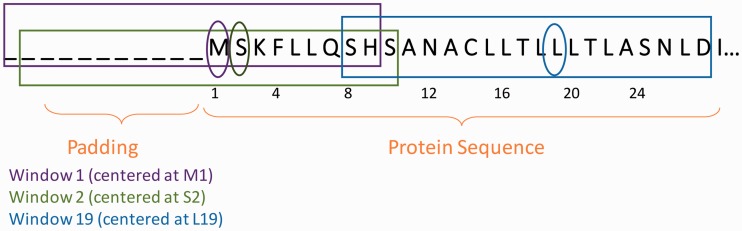
Window extraction and padding at the N-terminal of a protein. Initial Methionine is indexed 1. Each window is of size 20, having a prefix of 11 AA and a suffix of 8 AA. Therefore, the N-terminus of the protein should be padded with 11 ‘dummy’ AA, while the C-terminus would be padded with 8. For each residue along the sequence of the protein, there will be a corresponding window centered at this residue.
- Windows may be filtered by rules such as extracting only windows centered on a K or R (in the case of CleavePred). They may also be filtered by additional criteria, such as similarity to other windows. In CleavePred we removed duplicated windows that had 11 identical residues (the putative site flanked by five residues on each side), in order to remove redundancy and make the task more challenging.
- Sequence-based features are extracted for individual windows, creating fixed-length feature vectors. Additional features may be added from external sources, notably 2D structure, PSSM profiles, solvent accessibility and disorder, via the SCRATCH (26) and DISOPRED3 (15) toolkits.

Figure 1 illustrates the workflow of ASAP. The core of the framework is the ‘Window & feature extraction’ stage, comprised of the following sub-steps:

- Fixed-length overlapping windows are extracted, with each window becoming a sample in the training dataset. In the case of CleavePred, we extracted windows containing the 11 residues preceding (N-terminal wise) the putative site, and the eight residues after the site. For CleavePred, the label of each window is whether or not (1 or 0) its putative site residue is a PC cleavage site. ASAP is intended to solve any residue-level discrete binary classification problem (see Figure 2 for a concrete example of window extraction).

### ASAP features

ASAP supports multiple categories of features that are easily extendable. Most features are extracted by ASAP directly from the provided protein sequence, without relying on any external tools or databases. Exceptions are the optional features from external predictors (see below), and PSSM entropy which is derived from the PSSM profile. We refer the readers to the API for details, available at https://github.com/ddofer/asap.

#### External features

ASAP supports (optionally) externally created features, including predictions made according to primary sequences. We currently support PSSM profiles, and predictions for secondary structure, solvent accessibility and disorder. PSSM profiles are generated using SCRATCH’s ProfilPRO. Secondary structure (3 state resolution) and discretized solvent accessibility (buried or exposed) were predicted using SSpro and ACCpro (27). Discretized disorder predictions are obtained using DISOPRED3.

#### Local positional features

These properties relate to each individual position in the sequence. Discrete properties are encoded using one-hot-encoding (OHE). These features are:

- Amino Acid (AA) identity/reduced AA
- Secondary structure
- Intrinsic disorder
- Solvent accessibility
- AA electric charge (±1 or 0)
- PSSM (frequency of each AA in the PSSM profile at a position)
- PSSM entropy

We elaborate briefly on reduced AA alphabets and PSSM entropy. The former is a low dimensional representation of the AA alphabet, where biophysically similar ‘letters’ are grouped together. We used a variant of the alphabet with a reduced alphabet of 15 letter groups, previously used in ProFET (17). This reduces the amount of features, making the predictor less sensitive to over-fitting while making it easier to identify insights from high-level features (e.g. clusters of large and charged AAs). For a window of size 20, this eliminates 100 (potentially interacting) features.

PSSM entropy can be seen as a measure for the divergence of a position’s profile from a background distribution. The more conserved a position is, the lower its entropy (28). The conservation score is calculated using the relative entropy formula, taking relative background frequencies into account:

$$rRelative\;entropy=\sum_{i=1}^{20}p_{i}\cdot log(\frac{p_{i}}{pb_{i}})$$

Where $p_{i}$ are the AA frequencies according to the PSSM profile at a specific position in our protein of interest, and ${pb}_{i}$ are the background frequencies in naturally occurring proteins. In CleavePred, we used the background frequencies of vertebrates (29).

For the Intrinsic disorder, we included the naïve FoldIndex method (30), which predicts disorder as a function of hydrophobic potential and net charge.

#### Contextual aggregated features

Local protein regions surrounding a site of interest might have distinct aggregate properties. Hence, for various local features, we extracted an aggregation (e.g. max, avg) over multiple consecutive positions. The following three regions were often taken: [1,X-N-1],[X-N,X + N],[X + *N* + 1,L] where X is the index of the putative cleavage site, L is the window length and N is a predefined parameter (e.g. 4) salient to the immediate ‘neighbourhood’ of a site.

For example, we anticipated that a modified/cleaved site would be more conserved compared to the surrounding sequence (31). We thus aggregated the PSSM entropy within these segments, taking for each segment the minimum, maximum and average.

#### Motif features

We integrated classic motif-based approaches as regular expressions. While lacking in precision, these motifs have excellent sensitivity, and can help augment the other features. Motifs for additional PTMs can easily be added by users, or extracted from online knowledge based resources such as ELM.

In CleavePred, we included the “Known motif” feature of dibasic sites, which can be described as: X-X-K-[K or R], X-X-R-R, R-X-X-[K or R], where X denotes any AA (32,33). We also included the Cysteine spacer motif (34).

Total occurrences of the known motif signature were tallied if adjacent to the putative cleavage site. The Cysteine spacer motifs were counted regardless of location.

#### Global biophysical features

We included global features measuring biophysical properties for the entire window (or the whole protein, in the case of length):

- Molecular weight (in Da)
- Protein length (in AA)
- pH(I): the isoelectric point
- Net charge at various pH(I)
- Aromaticity: the relative frequency of Phe, Trp, Tyr
- Instability index: an estimate for the stability of a protein *in vitro*
- GRAVY (Grand Average of Hydropathy): the AAs’ average hydropathy
- Aliphatic index: the relative volume occupied by aliphatic side chains (Ala, Val, Ile and Leu).

Most of these properties were based on the ExPASy proteomics collection (35,36).

#### Amino-acid scale features

AA propensity scales map each individual amino acid to a quantitative value representing physicochemical or biochemical properties. We used a variety of different knowledge-based potentials, including hydrophilicity, polarizability, average solvent accessibility in a rigid tripeptide (ASA), the TOP-IDP disorder propensity scale (37) and additional maximally independent derived scales (38,39).

Features derived from these scales include: (i) Averages for pre-defined segments (in the spirit of the ‘Contextual aggregated features’ section); (ii) sliding overlapping segment averages, for segments of varying sizes. A full list of scales is provided in our source code: ‘AAScales.py’.

### CleavePred datasets

Two datasets of proteolytic cleavage were used: (i) Datasets from NeuroPred (32,40,41). (ii) Sequences gathered from UniProtKB/Swiss-Prot (42). These are manually annotated sequences labeled ‘cleavage on a pair of dibasic residues’ and annotated as ‘propeptide’ or ‘peptide’. The sequences were filtered both at the whole-protein and window levels, to provide a non-redundant, more challenging collection, using the following procedure:

- Removal of predicted Signal peptides from the sequences.
- Redundancy removal within datasets and between them (training and test sets). Redundancy was reduced using both CD-HIT (43) and USEARCH (44) by setting the maximal similarity level to 60%.
- Removal of windows with identical 11 residues centered around the putative site.

CleavePred’s windows were also filtered as in previous work (40, 45, 46). In brief, sites were candidates for dibasic cleavage if they had a K or R at the putative cleavage site, were located at least four positions ‘away’ from the N or C terminals. In the case of identical windows with different labels, the ‘cleaved’ label was treated as the ground truth.

### CleavePred ML algorithm

We tested different models implemented with scikit-learn (47). The final model used by CleavePred is a hard voting ensemble using the mlxtend package (https://github.com/rasbt/mlxtend), combining:

- Support Vector Machine with a radial basis function kernel
- Random forest (decision tree ensemble)
- Logistic regression

Unary (zero variance) features were automatically removed. During cross-validation (CV), for each independent fold, features were filtered using univariate feature selection (ANOVA F-value, false discovery rate of q < 0.1).

### Model evaluation and testing

We trained ‘simple’ and ‘advanced’ CleavePred models. The simple model uses only sequence-based features, while the advanced model also uses features obtained by external tools (see ‘Methods—External features’ section). The models were trained on NeuroPred’s dataset which contained, after removal of redundancy (see ‘Methods—CleavePred datasets’ section), 238 sequences. Of these sequences, ASAP extracted 6002 relevant windows (centered on K or R residues, Figure 2), from which 4802 windows comprised the final (NeuroPred) training set after the removal of similar windows (See ‘CleavePred datasets’ section), with 786 (16%) cleavage sites.

Performance was evaluated twice: first, on the training and evaluation Neuropred data using a stratified multiple CV procedure with 10 folds. CleavePred models trained on the complete NeuroPred dataset were then further validated on the UniProt-based test set. The latter contained 327 proteins after redundancy reduction, with 3455 candidate sites/‘windows’, containing 671 positive cleavage sites. The simple and advanced models extracted 657 and 1352 features, respectively. After feature selection, these were reduced to 482 and 960 features. Features that failed to pass the univariate statistical test were removed for each fold independently.

## Results

### Performance

Performance is evaluated based on 10-fold CV validation performance on the NeuroPred dataset, and on an independent hold out test set (UniProt). We ensured that the training and test datasets are disjoint and dissimilar (see ‘CleavePred datasets’ section). Table 1 show the performance of CleavePred and the Known Motif (KM) model (48) on the NeuroPred dataset, as measured by the average CV evaluation performance.

**Table 1.** Performance of CleavePred models (simple and advanced) and the known motif (KM) model on the NeuroPred dataset

Metric	Simple CleavePred (%)	Advanced CleavePred (%)	KM model (%)	Mammal model (%)
AUC	80.42	76.75	74.78	81.38
Accuracy	89.87	88.68	71.55	77.02
Sensitivity	64.98	57.23	81.60	68.69
Precision	79.13	78.69	48.29	55.92
Specificity	95.87	96.26	67.85	80.08
F1-Score	71.36	66.26	60.67	61.65

Performance measured using CV (10-fold) on 4,802 windows/samples. AUC: Area under ROC curve.

Table 2 shows our performance compared to two state-of-the-art competing methods, the Mammal model (M) (48) and the KM model (both using the implementation provided by the NeuroPred website), on the hold-out UniProt test set.

**Table 2.** UniProt test-set performance

	Simple CleavePred (%)	Advanced CleavePred (%)	KM model (%)
AUC	88.17	89.08	82.56
Accuracy	93.48	94.40	77.57
Sensitivity	80.28	81.17	89.72
Precision	79.97	84.06	49.26
Specificity	96.07	96.99	74.18
F1-Score	**80.13**	**82.59**	63.60

Several conclusions can be drawn from the analysis shown in Tables 1 and 2:

- Our models are superior in most measures of performance, both on the UniProt test set and the NeuroPred dataset (Table 1, NeuroPred CV).
- Massive improvement is seen in precision (from 48–55% to 79–84%).
- The performance on the test set (from UniProt/SwissProt) is lower with respect to the NeuroPred set (10-fold cross-validation, CV, compare Tables 1 and 2). Recall that the test set is ‘noisier’ and may suffer from shortage in true positives due to lacking experimental validation. Furthermore, some proteins in the validation and test set appeared in the Mammalian model’s training data, giving it an unrealistic advantage for these cases.

### Informative top features

We used Scikit-learn’s recursive feature elimination with cross-validation (RFECV) with a random forest (49, 50) in order to identify top features in each of the four configurations (simple and advanced models over NeuroPred and UniProt datasets). This procedure iteratively fits a classifier on the dataset and eliminates the least-informative features according to this classifier (random forest in this case). We focused on subsets of selected features common to both datasets. We found 44 such informative features for the simple model and 192 for the advanced one, which account for 9% and 20% of the original respective sets of features.

We note that our ‘engineered’ features appear consistently, while classic positional features (e.g. AA at each position) were less effective. Exceptions are the R or K at the position prior to the cleavage site (position 11,12 in CleavPred window, Figure 2).

The features that are well outside the ‘classic’ cleavage motif's location are of special interest. These features probably mark the preference for disorder quite remote from the actual cleavage recognition site.

Various AA scales were effective, notably solvent accessibility (51), Atchley scales at positions 0–4 and 7–12, tripeptide flexibility, Hydrophobicity (hw) and TOP-IDP at positions 6 and 13–16. Global features were also important, including the amount of basic AA prior to the cleavage site, GRAVY, Aromaticity, Aliphaticness, net charge and the presence of a potential known motif (KM).

For a detailed explanation on feature descriptors, see https://github.com/ddofer/asap.

In terms of advanced features, the PSSM and entropy based features proved quite powerful, both positional and in aggregated segments (including the maximal entropy segment). The aggregated sums of exposed, buried or intrinsically disordered to either side of the site were also important.

It should be noted however that many of the features are highly correlated with each other, and therefore the choice of some of them on the expense of the others is somewhat arbitrary. It should also be stressed that this procedure was applied only for reporting the top features in this section, and it was not part of the actual training, validation and testing of the model.

### Annotating novel genomes with CleavePred

Many of the peptides activated by PCs are peptide cell modulators. These peptides were studies in mammals and insects and to a lesser extent other taxonomical branches. *C. elegans* is an important model for cell lineage and development. Therefore, peptides that function in signaling and communication between neurons were sought. Tens of such peptides were identified using MS and comparative genomics (52). Many of these identified peptides were used for training CleavePred.

We tested CleavePred as a cleavage sites predictor on poorly annotated genomes. To this end, we selected the draft genome of *Ascaris suum* (Pig roundworm) (53). We focused on the secreted proteome (i.e. proteins with a putative Signal peptide). Among the tested sequences, several had high probability cleavage sites.

**Figure 3.**
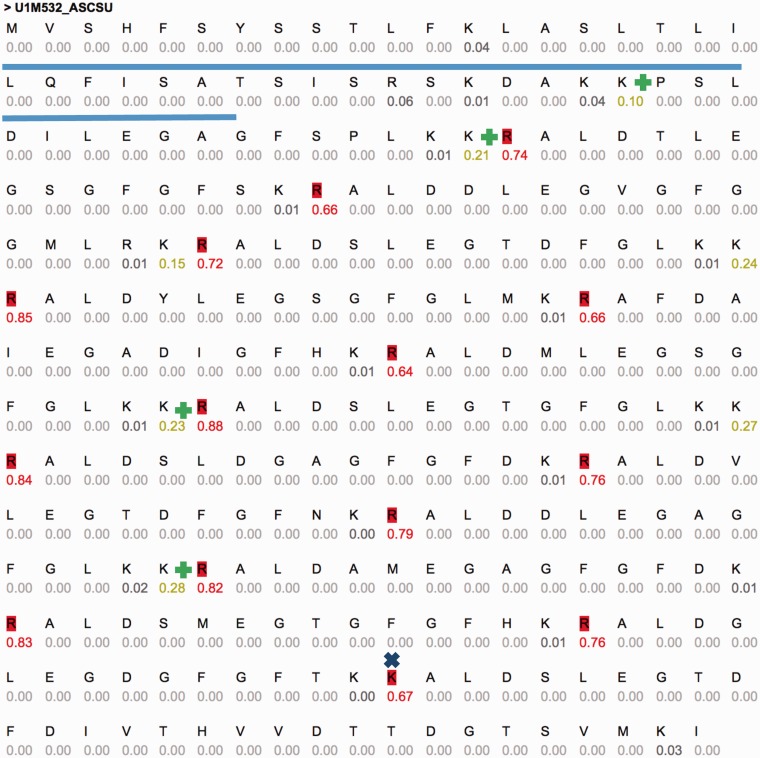
Example predictions using CleavePred’s website interface. Graphical view of CleavePred results for *Ascaris suum* genome (Pig roundworm, U1M532_ASCSU, 279 AA). While along the sequence there are 40 K/R residues, only 14 of them are predicted as cleavage sites (colored red, probability >0.5). Each residue is associated with its cleavage prediction. The repeated nature of the sequence is evident. The Signal sequence is underlined. X marks a missed cleavage site by ProP and additional cleavage sites according to NeuroPed (marked +).

One of these sequences is U1M532_ASCSU (Figure 3) that shows a repeated pattern of cleavage sites. Active peptides (14 high probability sites, 15 peptides, 14 AA each) were predicted using CleavePred. The confidence for the cleavage probability is high (0.64–0.88). Interestingly, identical cleavage pattern was found in other worms including *Toxocara canis* (Dog roundworm) and *Brugia malayi* (nematoda that infect humans). A similar organization of peptides was identified in crustacean Blue Crab (*Callinectes sapidus)* sinus gland. The repeated pattern (Figure 3) is common and was reported in Arthropods and insects (54). We conclude that CleavePred allows accurate prediction for active peptides is a wide range of poorly annotated genomes. ProP (19), A general convertase predictor identified 13 (of 14) sites. A discrepancy is observed at residue 251 of the sequence (GFGFTKK|AL, Figure 3, marked x). Other predictions of NeuroPred using default parameters are shown (Figure 3, marked +).

**Figure 4.**
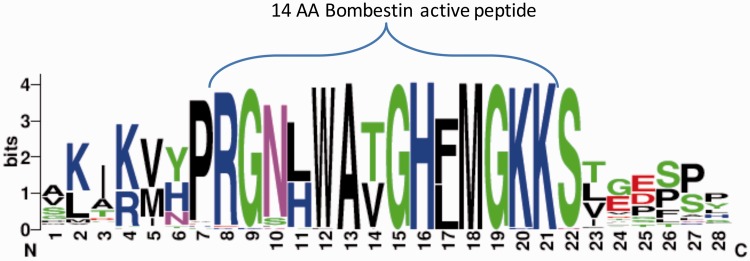
Bombestin putative peptides derived from Pfam PF02044 ‘uncharacterized’ proteins. Graphical view of the conserved region from 59 sequences named as ‘uncharacterized’ from Pfam’s model for Bombestin-like peptides (PF02044, 148 sequences). This set includes 23% of Neopterygii (new fins fish) and the rest are Amniota including representatives from reptiles, rabbit, elephant and more. For the majority of the sequences CleavePred identified the overlooked sites. Cleavage confidence at the N′-terminal sites was lower with respect to the cleavage site probabilities on the C′-terminal of the sequences (0.51–0.67 relative to 0.85–0.91, respectively).

We further tested the potential of ASAP-CleavePred pipeline to predict active peptides from ‘uncharacterized proteins’. We focused on Pfam's Bombestin-like peptide family that includes sequences from amphibian skin (27%) and mammalian (45%). We collected all 59 ‘uncharacterized’ proteins (Figure 4, Supplementary Data S1). We sought to identify cleavage sites regulating the production of short, potentially active peptides (8–14 AA) from the full proproteins. CleavePred identified paired cleavage sites for 24 of these sequences (at a probability threshold >0.5). For the rest of the sequences (35), only cleavage sites at the C’ terminal of the active peptides were predicted (Figure 4).

When the 59 uncharacterized sequences were analyzed with ProP with a relaxed setting for convertase cleavage sites prediction, only 11 high confidence sites were reported. None of ProP's results predicted two adjacent cleavage sites, thus no active peptides would have been predicted by this predictor in view of the 24 active peptides that were correctly predicted by CleavePred.

## Conclusion

In this study, we presented ASAP, a universal, generic, modular platform for extracting features and predicting local protein properties. ASAP is useful as a bioinformatics platform, allowing extensive analysis of new genomes and novel sequences. This generic framework can be applied to any residue-level problem. In our tutorial, (https://github.com/ddofer/asap/-wiki/Getting-Started:-A-Basic-Tutorial), we demonstrate the usability of ASAP in approaching biological problems and obtaining non-trivial results ASAP (i.e. in minutes). In the tutorial, we also demonstrate its use on another biological task of predicting phosphorylated serine. While feature engineering, fine-tuning and parameter optimization are always important, we suggest that ASAP is suited as an entry point for a wide range of prediction tasks.

We combined naive features, feature engineering (e.g. aggregated features), and simple ‘rule based’ patterns (i.e. the canonical ‘known motif’) (32). This combined approach outperformed the state-of-the-art results substantially. Our approach also supports integration of external properties such as structure. This provides superior performance to either individual method.

Analyzing the results from ASAP pipeline on CleavePred feature selection indicates that regions outside of the ‘canonical’ known motif itself affect whether a putative site is actually cleaved or not. We note our unexpected minor and sometimes negative (in terms of sensitivity) effects of adding structural features to the model, though adding just PSSM based features did provide a net benefit (Table 2).

We presented the power of ASAP towards the specific challenge of precursor protein proteolytic cleavage prediction (CleavePred). The number of substrates of processing enzymes in mammals is broader than anticipated. General convertase enzymes (PCs) regulate many pathways including lipid homeostasis, neoplastic and infectious diseases (55), as such PCs are attractive targets for therapeutics (56). For this task, we used a more challenging training and validation set and reported the results on a novel test set (Table 2).

We attribute the superior performance and usability of our results to the feature engineering at the heart of ASAP. CleavePred is extremely fast, and suitable for scanning multiple genomes. Due to the high cost of pursuing false-positives experimentally, the precision of CleavePred allows focus on only high-confidence candidates for further validation. Recall that CleavePred is suitable for any organisms and the performance is superior to models trained only on specialized subsets (e.g. mammal-model; Table 2). CleavePred provides highly confident prediction for a diverse collection of organisms (Figure 4). The generality of CleavePred in view of taxonomical coverage distinguish it from other prediction efforts trained only on selected taxa (e.g. Drosophila, humans).

CleavePred is accessible via a web interface at http://protonet.cs.huji.ac.il/cleavepred.

ASAP and CleavePred are free, open source (https://github.com/ddofer/asap), and come with a simple and well-documented Python API.

## Supplementary data

Supplementary data are available at *Database* Online.

## Funding

The project is partially supported by ELIXIR Accelerate grant (as part of the ELIXIR-IL). This research was partially funded by H2020, ELIXIR-EXCELERATE grant.

*Conflict of interest*: None declared.

## Supplementary Material

Supplementary Data
