# Supplementary material for: ASAP: a machine learning framework for local protein properties
Source: Database (Oxford). 2016 Oct 1;2016:baw133. doi: 10.1093/database/baw133 (PMC5045867; doi:10.1093/database/baw133)
Supplement: Supplementary Data [file supp_2016_baw133_index.html]

Supplementary Data 

# ASAP: a machine learning framework for local protein properties

## Supplementary Data

files

- Supplementary Data - docx file
