# Supplementary material for: ASAP: a machine learning framework for local protein properties
Source: Database (Oxford). 2016 Oct 1;2016:baw133. doi: 10.1093/database/baw133 (PMC5045867; doi:10.1093/database/baw133)
Supplement: Supplementary Data [file supp_baw133_ASAP-final-Supplementary_Data_1.docx]

# Supplementary Data 1

# List of 59 Pfam “uncharacterized” sequences of Bombestin

H2QEL4

H2QA05

M3WUJ9

G3WTK8

G3WHY0

F7E042

F7DYM4

G1NYT2

G1M9S0

G1TEL7

G1SWS1

H0VMK5

I3JLE7

G1MQK3

B3DFU2

A0MAR5

Q1MT60

H2NPG8

G3QJM8

M3XKD6

M4ARS9

H2SFV6

G1M601

H0W595

I3LZ49

H0Z347

F7DIJ3

F7IC55

M3YYG7

M3YW01

W5PUI9

H0XTF5

F6QB63

F6X8X8

G3T507

G3T1M6

G1RKG4

G1RAD3

J9NU40

G3S1K3

G3RFA9

D2HT64

G1P5L3

G1Q1B7

I3MKE8

I3NEH6

W5KQX4

W5LBR7

W5KLR3

V8NXR9

V8NUQ7

U3K5M0

U3K576

I3J2T7

H3BWV3

M3ZLC4

M3ZQA0

W5N1Y3

W5NBQ7
